# On the (lack of) association between theory of mind and executive functions: a study in a non-clinical adult sample

**DOI:** 10.1038/s41598-020-74476-0

**Published:** 2020-10-14

**Authors:** Marialaura Di Tella, Rita B. Ardito, Federico Dutto, Mauro Adenzato

**Affiliations:** 1 Department of Psychology, University of Turin, Turin, Italy; 2 Department of Neuroscience “Rita Levi Montalcini”, University of Turin, via Cherasco, 15, 10126 Turin, Italy

**Keywords:** Psychology, Human behaviour, Social neuroscience, Neuroscience, Cognitive neuroscience, Intelligence

## Abstract

We investigated in a sample of non-clinical adults the association between Theory of Mind (ToM) and Executive Functions (EFs), that is the set of skills that allow people to control and modulate lower-level cognitive processes in order to produce appropriate behaviour. To this aim, we assessed both affective (i.e., understanding other people’s emotions and feelings) and cognitive (i.e., understanding others’ beliefs and intentions) ToM, as well four subcomponents of EFs, that is Updating, Shifting, Inhibition, and Access. The association between ToM and non-verbal fluid intelligence, verbal reasoning, and cognitive estimation abilities was also investigated. Eighty-one healthy participants were recruited, and a set of psychometrically validated tests was administered. Multiple regression analyses were run to assess significant predictors of ToM performance when potentially confounding predictors (sociodemographic variables) were controlled for. Results showed a lack of association between affective/cognitive ToM and EFs, whereas non-verbal fluid intelligence for cognitive ToM and verbal reasoning for affective ToM were found to be significant predictors of ToM performance. These results represent a contribution toward a deeper understanding of the ToM-EFs relationships and highlight the importance of broadening the analysis of these relationships to the role played by other domain-general functions in both affective and cognitive ToM.

## Introduction

The term Theory of Mind (ToM) refers to the abilities to understand and attribute mental states, such as desires, intentions and beliefs, to others. These abilities gradually evolve during the lifetime and allow individuals to understand adequately the social world and to interact effectively with other people^1–4^.

ToM abilities have been traditionally distinguished in first and second order processes based on the complexity of ToM skills required for the understanding of another person’s false belief^5^. In more recent years, evidence from different lesion studies has led authors to propose a further differentiation between affective ToM, that is the ability to detect and experience others’ feelings and emotions, and cognitive ToM, that is the ability to identify others’ beliefs or intentions^6,7^.

An open issue concerns the association between ToM abilities and the high-level cognitive processes known as executive functions (EFs). The majority of studies have tried to address this relationship in patients with different clinical conditions^8–13^. However, only a limited number of studies (e.g.,^14–16^) have attempted to investigate the association between ToM and EFs in non-clinical adult populations, by going beyond a simple correlational analysis and trying instead to investigate the possible predictor role of EFs in explaining ToM performance.

EFs refer to the set of skills that allow people to control and modulate lower-level cognitive processes in order to produce appropriate behaviour^17^. These abilities are therefore considered to be essential for an independent everyday life functioning and the implementation of adequate social interactions^18,19^. During the last two decades, different classifications have been proposed to distinguish the main subcomponents of EF domain. The model elaborated by Miyake et al.^20^ and subsequently revised by Fisk and Sharp^21^ identified four main types of operations: Updating, Shifting, Inhibition, and Access. *Updating* is related to working memory and requires monitoring and coding information as well as replacing old non-relevant information with new relevant one. *Shifting* concerns the ability to engage and disengage attention from different sub-tasks. *Inhibition* implies holding back automatic or preponderant responses and is considered to be a key component in planning abilities. *Access* is involved in verbal fluency tasks and mediates access to representations in long-term memory.

The relationship between ToM abilities and EF processes has been conceived differently over time. Original theorisations considered ToM as a circumscribed domain-specific process, independent of other cognitive domains, included EFs^1,22,23^. More recent evidence has suggested, instead, that understanding others’ mental states may involve both modular processes and domain-general processes such as EFs^24,25^. In particular, lower-level cognitive mechanisms (e.g., detection of gaze direction and joint attention), on which the adequate development of ToM skills is based, have been thought to be domain specific abilities, while higher-order ToM abilities, involving interpreting and associating information as well as hypothesising, have been suggested to be more depended on domain-general cognitive processes such as EFs^25^.

Another aspect worthy of consideration when assessing the relationship between ToM and EFs, is the different and specific associations that may be found among the two ToM dimensions (viz., affective and cognitive ToM) and the various subcomponents in which EFs have been distinguished. Indeed, previous evidence in clinical populations seems to show that the relationship between EFs and ToM could be limited to the cognitive dimension of ToM^12,26,27^, with non-significant associations between affective ToM tasks and EFs measures^9,28^. However, divergent evidence has been reported. For instance, Grosse Wiesmann et al.^29^ by means of tract-based spatial statistics and probabilistic tractography demonstrated that in early childhood the emergence of false belief understanding (a crucial test for cognitive ToM) is correlated with white matter maturation in a set of brain regions (including the medial prefrontal cortex) independently of the co-developing EF abilities. Vice versa, some studies reported correlations between EFs and affective ToM. For example, Yildirim et al.^30^ found that set shifting and inhibition were significantly correlated with affective ToM assessed by means of the Reading the Mind in the Eyes Test (the most used test to assess affective ToM, see below) among a group of young adults ranging from 18 to 28 years.

Based on these premises, the main aim of the present study was to further shed light on the association between ToM and EFs in a sample of non-clinical adult individuals. Particularly, we aimed to assess if the four above-mentioned subcomponents of EF domain, identified by Fisk and Sharp^21^, could be differently related to the affective vs. cognitive dimensions of ToM. Furthermore, since some studies have showed significant relationships between ToM functioning and other cognitive abilities^31–35^, the association between ToM and non-verbal fluid intelligence, verbal reasoning, and cognitive estimation abilities was also evaluated.

## Results

### Descriptive analyses

Data on age, educational level, ToM, EFs, non-verbal fluid intelligence, verbal reasoning, and cognitive estimation of the total sample are presented in Table 1.

**Table 1:** Data on age, educational level, ToM, EF, non-verbal fluid intelligence, verbal reasoning, and cognitive estimation measures of the total sample (*N* = 81).

	Mean	SD	Range
Age (years)	21.23	1.89	19–27
Educational level (years)	15.07	1.27	13–18
**Theory of Mind**			
RMET	26.49	3.14	14–33
Strange stories—ToM score	12.37	2.29	5–16
Strange stories—Physical score	10.47	2.58	5–15
**Executive functions**			
TMT B^a^	120.49	20.09	86.84–174.55
DS B^b^	3.72	0.98	1.38–6.38
FAS	11.86	2.56	4–16
ToL	29.14	3.49	18–41
**Other cognitive abilities**			
SPM	30.24	3.65	15.50–36.25
VRT—total	80.74	5.87	68.30–92.87
VRT—absurdities	11.04	2.12	3.46–13.48
VRT—intruders	12.09	1.31	7.11–13.11
VRT—relationships	12.32	1.08	9.30–13.30
VRT—differences	12.04	1.45	8.68–13.68
VRT—idiomatic	11.00	1.91	4.53–14.53
VRT—family relations	10.31	2.59	0.36–21.39
VRT—classifications	12.20	1.60	7.01–13.49
CET—total deviation scores	15.37	3.17	8.20–21.20
CET—very extreme responses	3.79	2.09	0–10

*SD* standard deviation, *RMET* reading the mind in the eyes test, *ToM* theory of mind, *DS B* digit span backward, *TMT* trail making test, *FAS* verbal fluency, *ToL* tower of London, *SPM* standard progressive matrices, *VRT* verbal reasoning test, *CET* cognitive estimation task.

^a^The unit of measure for the TMT B is in seconds.

^b^The DS B original scores have been corrected for age and educational level, in accordance with the indications provided by Orsini et al.^69^.

As far as the other sociodemographic characteristics are concerned, the majority of participants were ‘never-married’ (77, 95.1%), followed by ‘cohabitant’ (3, 3.7%) and ‘married’ (1, 1.2%). Regarding occupation, most participants were students (78, 96.3%), whereas 3 (3.7%) were employed.

### Correlation analyses

The results of the correlation analyses are presented in Table 2.

**Table 2:** Pearson (*r*) or point-biserial (*r*_pb_) correlations among sociodemographic variables, ToM, EF, non-verbal fluid intelligence, verbal reasoning, and cognitive estimation measures (*N* = 81).

	RMET	ToM strange stories	Physical strange stories
Age (*r*)	.010	.067	.111
Educational level (*r*)	.091	.081	.020
Gender (*r*_pb_)	− .121	.096	− .070
TMT B (*r*)	− .132	− .256	− .047
DS B (*r*)	.076	− .024	.094
FAS (*r*)	− .120	.018	− .034
ToL (*r*)	− .109	.192	.266*
SPM (*r*)	.006	.387**	.100
VRT—total (*r*)	.272*	.317**	.301**
CET—total deviation scores (*r*)	− .105	− .109	− .046
CET—very extreme responses (*r*)	− .058	− .206	.039

*RMET* reading the mind in the eyes test, *ToM* theory of Mind, *TMT* trail making test, *DS B* digit span backward, *FAS* verbal fluency, *ToL* tower of London, *SPM* standard progressive matrices, *VRT* verbal reasoning test, *CET* cognitive estimation task.

* *p* < .017; ** *p* < .01.

As far as the affective ToM is concerned, higher scores on the RMET were positively correlated with the total score of the VRT.

Regarding the cognitive dimension of ToM, positive and significant correlations were detected between the ToM SS scores and both the SPM scores and the total score of the VRT. Similarly, higher scores on the Physical SS were found to be positively associated with the ToL scores and the total score of the VRT.

No significant correlations were found between ToM measures and sociodemographic variables (i.e., age, educational level, and gender).

### Multiple regressions

To investigate whether EFs and other cognitive abilities measures were significant predictors of ToM measures, three multiple regression analyses were performed. The RMET was used as a dependent variable in the first regression, while the ToM and Physical SS scores were used in the second and third regression, respectively. Since the variables of age, educational level, and gender did not correlate with the dependent variables, they were no longer included in the regression analyses. Therefore, the final regression models included only EFs and other cognitive measures as predictor variables, and they were entered simultaneously into each model.

With regard to the RMET, the model was statistically significant, R^2^ = .07, *F*(1, 79) = 6.320, *p* = .014; adjusted R^2^ = .06 and the total score of the VRT significantly contribute to explanation of the RMET score (*β* = .272, *p* = .014) (Table 3).

**Table 3:** Multiple regressions predicting ToM tasks scores from EF, non-verbal fluid intelligence, verbal reasoning, and cognitive estimation measures (*N* = 81).

Predictors	B	β	t	95% CI	R^2^	Adj R^2^	F
**RMET**							
Model					0.07	0.06	6.320*
VRT—total	0.146	0.272	2.514*	0.030; 0.261			
**ToM strange stories**							
Model					0.40	0.17	7.781**
SPM	0.191	0.304	2.485*	0.038; 0.344			
VRT—total	0.060	0.153	1.252	− 0.035; 0.155			
**Physical strange stories**							
Model					0.18	0.16	8.606**
ToL	0.224	0.302	2.932**	0.072; 0.376			
VRT—total	0.147	0.334	3.239**	0.057; 0.237			

*RMET* reading the mind in the eyes test, *CI* confidence interval, *ToM* theory of mind, *SPM* standard progressive matrices, *ToL* tower of London, *VRT* verbal reasoning test.

* *p* < .05; ** *p* < .01.

Whereas as far as the ToM SS are concerned, the full model of cognitive measures (SPM and total score of the VRT) to predict cognitive ToM was statistically significant, R^2^ = .17, *F*(2, 78) = 7.781, *p* = .001; adjusted R^2^ = .15. In this case, only the SPM (*β* = 0.304, *p* = .015) was found to be a significant contributor of the model (Table 3). Following the suggestion of one of the reviewers, we have also verified if any changes in the regression model could occur using an α = .05/2 (i.e., including in the analysis the ToM SS score but not the Physical SS score). Despite an additional significant correlation that emerged between the ToM SS and the TMT B (*p* = .021), the only significant predictor in the final regression model was still found to be the SPM (*β* = 0.277, *p* = .031).

Finally, regarding the Physical SS, the full model of cognitive measures (ToL and total score of the VRT) to predict physical passages was statistically significant, R^2^ = .18, *F*(2, 78) = 8.606, *p* < .001; adjusted R^2^ = .16. Both ToL (*β* = 0.302, *p* = .004) and the total score of the VRT (*β* = 0.334, *p* = .002) were found to be significant predictors in the model (Table 3).

In all regression analyses, the statistical factor of tolerance and VIF showed that there were no interfering interactions between the variables.

## Discussion

The present study mainly aimed to shed light on the association between ToM and EFs in a sample of non-clinical adult individuals. To reach this goal we assessed both affective and cognitive ToM, as well as the four different subcomponents of EF domain identified by Fisk and Sharp^21^, that is Updating, Shifting, Inhibition, and Access. Furthermore, since significant relationships between ToM functioning and other cognitive abilities have been described, the association between ToM and non-verbal fluid intelligence, verbal reasoning, and cognitive estimation abilities was also investigated.

Results of correlation analyses showed the presence of no significant association between EF measures and either the affective (i.e., RMET) or the cognitive (i.e., mental SS score) dimensions of ToM. A significant association was only detected between the ToL (i.e., Inhibition subcomponent of EFs) and the Physical SS score. A different pattern of results was found for the other cognitive abilities measures. Indeed, the results of the regression analyses showed a significant predictor role of the VRT total score in explaining the RMET, whereas the SPM was found a significant predictor of the mental SS test. Finally, the VRT total score, together with the ToL, significantly predicted the Physical SS score.

Our findings are partly convergent and partially divergent with the few previous studies (e.g.,^14–16^) that have investigated the association between ToM and EFs in non-clinical young adults, going beyond a simple correlational analysis but specifically searching for the possible predictor role of EFs in explaining ToM performance. Indeed, a lack of association between affective ToM and EFs was also found by Bottiroli et al.^14^ that did not find such association between the affective component of the Faux Pas task (a task in which a character commits a social gaffe by saying something s/he shouldn’t have said) and two EFs tasks used to assess inhibition and updating (i.e., the Hayling task and the working memory updating task, respectively). With regard to cognitive ToM, these authors have found that the performance to the cognitive component of the Faux as task is not predicted by the inhibition task and is associated with only one of the four subscales of the working memory updating task. Similar results were found by Fischer et al.^15^ that did not find an association between both the affective component of the Yoni task and the RMET, and three EFs tasks assessing inhibition, working memory and auditory attention, but found an association between the cognitive component of ToM, as assessed by the SS stories, and a composite variable representing the scores to all the EFs tasks employed in their study (unfortunately, Fischer et al.^15^ do not provide the results concerning the single associations between cognitive ToM and each of the EFs tested). Finally, an association between affective ToM, as assessed by the visual component of the Cambridge Mindreading Face-Voice battery (CAM), and two inhibition conditions, one emotional, another non-emotional, was found by Mahy et al.^16^. The CAM is a task in which adult actors express dynamic complex emotions in the face and torso, and participants have to select one of four adjectives presented that best describes the emotion of the person in the video. During the viewing of each video clip, Mahy et al.^16^ auditorily presented four emotion words (emotional inhibition) or two neutral nouns and two neutral verbs (non-emotional inhibition) and instructed participants to ignore these words. The authors found that both the emotional and non-emotional inhibition conditions resulted in lower judgment accuracy than a control condition of no inhibition. However, contrary to their hypothesis, there was no difference in ToM judgment accuracy between the emotional and non-emotional inhibition conditions. Interestingly, Mahy et al.^16^ recognize that their results can best be described in the terms previously proposed by Newton and de Villiers^36^, that is language is necessary in complex ToM tasks, even when the ToM task is nonverbal, an observation that fits with our finding concerning the predictor role of the VRT total score in explaining the RMET.

A separate note deserves our result showing that inhibition, as assessed by the ToL, together with the VRT total score, significantly predicted the Physical SS score. With regard to the Physical SS, we have decided to include in our study also this part of the task, along with ToM SS, as those stories can help to differentiate text comprehension abilities, which are required for the correct understanding of both types of passages, from ToM abilities, necessary for the adequate comprehension of ToM SS only. We did not have an a priori expectation about the association between Physical SS and EFs but our finding seems to confirm previous studies^37,38^ that have questioned the fact that the Physical SS are well matched with the ToM SS, and indeed, as Brewer et al.^39^ pointed out, the Physical SS have never been subjected to a rigorous psychometric item analysis. We suppose that this could explain the different associations we found in the Physical SS and in the ToM SS.

Our findings, which show the absence of association between EFs and both affective and cognitive ToM, are in line with both the original theorisations that considered ToM as a domain-specific system independent of EFs^1,22,23^, and neuropsychological findings showing that executive functioning and ToM abilities are dissociable^9–11,40–48^. For example, Bird et al.^44^ reported the case of a patient, G.T., suffering an exceptionally rare form of bilateral infarction in the brain territory supplied by the anterior cerebral artery, that is, the medial frontal lobes bilaterally. Despite a marked dysexecutive syndrome resulting from her brain damage, G.T. showed unimpaired affective and cognitive ToM abilities. Furthermore, Aboulafia-Brakha et al.^18^ conducted a systematic review of 24 studies to offer a global view of the pattern of association and dissociation between ToM and EFs performances in patients with acquired neurological disorders. Interestingly, these authors categorized executive tasks used in the studies they reviewed according to the model of EFs proposed by Fisk and Sharp^21^, that is, the model we used as a guide in the present study. These authors concluded that the pattern of association and dissociation they found suggests that ToM abilities and EFs might share common mechanisms but still need to be considered distinct functions.

While our findings on the relationship between cognitive ToM and EFs are in line with most of the neuropsychological literature they are divergent with the majority of the developmental literature. Indeed, studies conducted in child samples have often revealed significant associations between ToM and EFs^49–53^. It has been suggested that the reason why children’s belief reasoning is related to executive functioning is because EFs are an integral part of the mature capacity for belief reasoning in adults^54^. However, recent studies adopting a developmental cognitive neuroscience perspective call into question early assumptions according to which EFs are a necessary prerequisite for the development of cognitive ToM abilities. For example, Richardson et al.^55^ using fMRI show that in typically developing children (3–12 years) the ability to solve cognitive ToM tasks relies on the same brain network recruited when adults reason about others’ mental states (i.e., medial prefrontal cortex, temporoparietal junction, and precuneus) and that over time this network gradually becomes more integrated and distinct from other networks. More importantly, combining white matter measures acquired by means of tract-based spatial statistics and probabilistic tractography with behavioural performance in false belief tasks, it has been recently demonstrated that the developmental breakthrough in false belief understanding in 3- and 4-year-old children is associated with age-related changes in local white matter structure in temporoparietal regions, the precuneus and medial prefrontal cortex, and that these effects are independent of co-developing EFs^29,56^. As suggested by Grosse Wiesmann et al.^56^, these findings are inconsistent with the view that young children only fail explicit ToM tasks due to extrinsic executive demands of these tasks because, in that case, brain regions involved in EFs should be related to success in the tasks.

To date the balance of converging evidence coming from developmental and cognitive neuroscience suggests that separable mechanisms underlie ToM and EFs, with shared mechanisms for domain-general processing that support both abilities^57^. Our findings are in line with this view and suggest the lack of association between ToM and EFs, and the supporting role played by other domain-general functions in both affective and cognitive ToM. Indeed, the multiple regression analyses we performed showed no direct relationships between ToM and EFs but a significant predictor role of non-verbal fluid intelligence for cognitive ToM performance and of verbal reasoning for affective ToM performance. These results are in line with the few previous studies that directly investigated the relationships between affective and cognitive ToM and these two cognitive abilities. For example, concerning non-verbal fluid intelligence, Rakoczy et al.^35^ found that ToM performance in healthy adults, as assessed inter alia by the Happe’s Strange Stories, is related to processing speed (generally considered one of the best proxies for fluid intelligence) but not to EFs. Similarly, Coyle et al.^31^ using structural equation modelling found a significant correlation between ToM and a factor of intelligence assessed by using a college admissions test strongly correlated with the non-verbal Raven’s Matrices, suggesting that this factor might facilitate the ability to make complex inferences in everyday life including the inferences involved in understanding the mental states of others. Furthermore, Giovagnoli et al.^58^ showed that after an anterior temporal lobectomy the ability to understand others’ mental states in patients with epilepsy was related to Raven’s Coloured Progressive Matrices performance, thus supporting the view that fluid intelligence contributes to ToM.

Finally, concerning the relationship between verbal reasoning and affective ToM, Peterson and Miller^59^ have demonstrated, by means of the Vocabulary subtest of the Wechsler Abbreviated Scales of Intelligence, that in young adult verbal IQ alone accounts for almost 25% of the variance in RMET performance, and Baker et al.^60^ conducted a meta-analysis providing evidence of a small but robust and stable significant relationship between verbal reasoning and the RMET performance.

The present study has some limitations that we would like to acknowledge. First, we adopted a cross-sectional design, which does not permit us to draw firm conclusions about the causality of the emergent relationships. Secondly, even though we enrolled an adequate number of participants, further studies, recruiting larger samples of participants, are needed to confirm the current results. Finally, the present study used only two ToM tasks and examined EFs in relation to ToM abilities only in a sample of young adults. In order to ascertain the generalizability of the present findings, future studies should be carried out using further ToM tasks that assess both cognitive and affective ToM (e.g., the Yoni task^61^ or the Movie for the Assessment of Social Cognition^62^) and recruiting healthy participants with heterogeneous sociodemographic characteristics.

## Conclusion

To the best of our knowledge, to date no studies have investigated at the same time in non-clinical adults the relationships among the two ToM dimensions (viz., affective and cognitive) and the four different subcomponents of EFs (viz., Updating, Shifting, Inhibition, and Access), together with other cognitive abilities, such as non-verbal fluid intelligence, verbal reasoning, and cognitive estimation. Despite the limitations described above, the findings reported in the present study represent a contribution toward a deeper understanding of the ToM-EFs relationships. Indeed, we showed a lack of association between affective/cognitive ToM and EFs, and a supporting role of non-verbal fluid intelligence for cognitive ToM performance and of verbal reasoning for affective ToM performance.

Previous studies that examined the ToM-EFs relationships have often omitted to investigate the contribution to ToM functioning of cognitive abilities other than EFs. Here we highlighted the importance of broadening the analysis of these relationships to the role played by other domain-general functions in both affective and cognitive ToM.

## Methods

### Participants and procedure

Eighty-one healthy participants were recruited with the following inclusion criteria: more than 18 years old, more than five years of educational level, adequate knowledge of the Italian language, and no history of a neurological or severe psychiatric disorder. The sample was equally divided between men (41, 50.6%) and woman.

After giving their agreement to take part in the study, participants were asked to indicate sociodemographic (i.e., age, gender, educational level, marital status, and occupation) and clinical information (i.e., history or presence of psychiatric or neurological disorders), and to complete a series of performance-based measures (i.e., task assessing affective and cognitive ToM, EFs, non-verbal fluid intelligence, verbal reasoning, and cognitive estimation).

The study was approved by the University of Turin ethics committee (Prot. n. 10036) and was conducted in accordance with the Declaration of Helsinki. All the participants gave their written informed consent to participate in the study.

### Measures

#### Theory of mind assessment

##### Strange stories test

The Italian translation of the Strange Stories (SS) test has been used for the assessment of cognitive ToM^63–66^. It consists of two types of short stories: ToM stories and physical stories. The eight ToM stories require the participants to infer characters’ mental states and concern double bluff, mistakes, persuasion, and white lies (two examples for each story type). Conversely, the eight physical control stories do not involve mental states but require participants to make global inferences that go beyond what was explicitly mentioned in the text.

Each story is followed by a question assessing the ability to infer the characters’ thoughts and feelings, for ToM passages, while for non-mental-state stories, to understand, for example, physical causation.

The total score for both ToM and physical stories ranges from 0 to 16, with higher scores indicating better performance.

##### Reading the mind in the eyes test

The Italian translation of the Reading the Mind in the Eyes Test (RMET) was employed to assess the ability to represent other people’s affective mental states^67,68^. In the test, the experimenter presents a set of 36 photographs of the eye region of various human faces. Participants viewed 36 photographs of the eye region of various human faces and were required to choose one of four words, using the criterion of which word best describes the mental state of the person depicted in the photograph. Participants have to put themselves into the mind of another person to recognise his or her complex mental state. They had unlimited time to decide and a glossary was provided. A score of 1 is given for every correct answer, with a maximum possible score of 36.

#### Executive functions and other cognitive abilities assessment

As far as EFs are concerned, we investigated the four subcomponents into which EFs have been divided according to the models of Miyake et al.^20^ and Fisk and Sharp^21^, using four specific tests: (1) the Digit Span-Backward (DS B)^69^, which requires the participant to repeat the numbers in the reverse order of that presented by the examiner, has been used for Updating; (2) the TMT B^70^, which requires the individual to draw lines sequentially connecting encircled numbers and letters (e.g., 1, A, 2, B, 3, C, etc.) alternately, has been employed for Shifting; (3) the Tower of London (ToL)^71,72^, which requires the participant to move perforated balls, placed in a certain configuration on a particular structure, until the configuration shown by the experimenter is reached, has been used for Inhibition; (4) the verbal fluency (FAS)^73,74^, which requires the participant to produce as many unique words as possible starting with a given letter in 1 min, has been employed for Access. In addition, we administered the Standard Progressive Matrices (SPM)^75^, which involve completing a pattern or figure with a part missing by choosing the correct missing piece among six alternatives, for the assessment of non-verbal fluid intelligence; the Verbal Reasoning Test (VRT)^76^, which is made up of seven subtests (i.e., absurdities, intruders, relationships, differences, idiomatic expressions, family relations, and classifications) that assess different aspects of verbal reasoning, for the evaluation of verbal reasoning in young adults; and the Cognitive Estimation Task (CET)^77,78^, which requires complex processes such as reasoning, the development and application of appropriate strategies, response plausibility checking and general knowledge and numeracy, for the investigation of cognitive estimation abilities.

### Statistical analyses

The statistical analyses were carried out with the Statistical Package for Social Science, version 25.0 (IBM SPSS Statistics for Macintosh, Armonk, NY, USA: IBM Corp.).

Indices of asymmetry and kurtosis were used to test for normality of the data. Values for asymmetry and kurtosis between – 1 and + 1 were considered acceptable in order to prove normal univariate distribution. All variables resulted normally distributed.

First, Pearson (*r*) or point-biserial (*r*_*pb*_) correlations were computed to evaluate the possible relationships between sociodemographic variables (age, educational level, and gender), ToM (RMET and SS), EFs (DS B, TMT B, FAS, ToL), and other cognitive abilities (SPM, VRT, and CET) tasks. Bonferroni correction for multiple testing was applied (α = .05/3).

Secondly, multiple regression analyses were run to assess whether EFs, non-verbal fluid intelligence, verbal reasoning, and cognitive estimation were significant predictors of ToM performance when potentially confounding predictors (sociodemographic variables) were controlled for. ToM tasks were used as dependent variables. The predictor groups were entered into the regression model according to the following schema: sociodemographic variables (age, educational level, and gender), and cognitive abilities measures (EFs, non-verbal fluid intelligence, verbal reasoning, and cognitive estimation). The enter method was used to include the variables of the predictor groups. To avoid unnecessary reductions in statistical power, predictor variables were included in the regression models only when they were significantly correlated with the dependent variables (*p* < .017). With regard to the VRT, we entered in the regression model only the total score, in order to avoid multicollinearity problems.

Collinearity was assessed through the statistical factor of tolerance and Variance Inflation Factor (VIF).

## Data Availability

The datasets generated during and/or analysed during the current study are available from the corresponding author on reasonable request.
